# Failure of fixation of trochanteric femur fractures: Clinical recommendations for avoiding Z-effect and reverse Z-effect type complications

**DOI:** 10.1186/1754-9493-5-17

**Published:** 2011-06-22

**Authors:** Robinson Esteves Santos Pires, Egídio Oliveira Santana, Leandro Emílio Nascimento Santos, Vincenzo Giordano, Daniel Balbachevsky, Fernando Baldy dos Reis

**Affiliations:** 1 Hospital Felício Rocho, Av. do Contorno 9790, 30110-060, Belo Horizonte, MG, Brasil; 2 Hospital Baleia, Rua Juramento 1464, Saudade, 30285-000, Belo Horizonte, MG, Brasil; 3 Serviço de Ortopedia e Traumatologia Prof. Nova Monteiro, Hospital Municipal Miguel Couto, Rua Mario Ribeiro 117, Gávea, 22430-160, Rio de Janeiro, RJ, Brasil; 4 Universidade Federal de São Paulo, Escola Paulista de Medicina, Rua Botucatu 740, 04023-900, São Paulo, SP, Brasil

## Abstract

**Background:**

Z-effect and reverse Z-effect are complications that arise from the surgical treatment of pertrochanteric fractures of the femur with proximal femoral nails (PFN) comprising two interlocking head screws. Such complications are induced by the migration of screws in opposite directions, which may lead to failure of the osteosynthesis.

**Findings:**

The paper describes three cases of pertrochanteric fractures that were treated with PFN with two interlocking screws that evolved to either Z-effect or reverse Z-effect. Literature-based explanations for this phenomenon are provided together with recommendations of how to avoid such complications.

**Conclusions:**

Although intramedullary fixation is an established method of treatment of femoral intertrochanteric and subtrochanteric fractures, the evolution of the procedure may include complications associated with the migration of the interlocking head screws. The occurrence of Z-effect and reverse Z-effect has not been completely elucidated, but the main causes of such complications are probably fracture fixation in varus position, severe medial comminution, inappropriate entry point of the nail and poor bone quality.

## Background

The incidence of fractures of the proximal femur has increased considerably over the last few decades as a consequence of the greater longevity of the population [1]. Femoral pertrochanteric fractures (FPF) typically occur in patients presenting diverse types of comorbidities and are associated with a high rate of mortality in the first year after the event [2].

In the present paper, we describe the complications observed in three patients arising from surgical treatment of FPF with proximal femoral nails (PFN) comprising two interlocking head screws that migrated in opposite directions (Z-effect and reverse Z-effect). The possible causes of these complications and relevant preventive methods are discussed.

### Case 1

**Figure 1:**
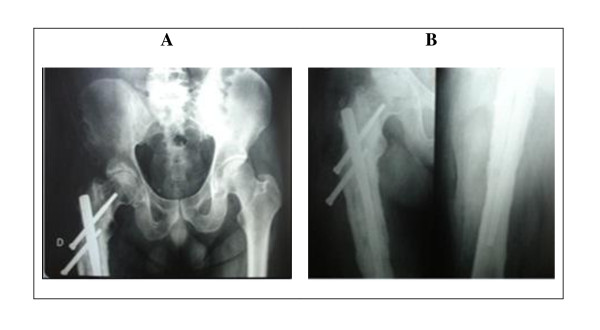
**Case 1 - Radiographic images of the pelvis in anteroposterior view (A), and the hip joint in anteroposterior and lateral view (B), showing the subtrochanteric fracture of the right femur consolidated in varus angulation**. The reduction in varus and the intense periosteal reaction caused by infection can be observed. The entry point of the nail is excessively lateral, and the interlocking screws are short and incorrectly positioned culminating in the lateral migration of the inferior screw (Z-effect).

A 44-year old male doctor (with no comorbidities), who had been injured in a car accident, presented subtrochanteric fracture (AO/OTA 32-B1) of the right femur and was submitted to osteosynthesis with PFN (Hexagon^®^, Campinas, SP, Brazil) one day after the trauma. The femur fracture was classified as level 3 on the abbreviated injury scale (AIS), whereas the injury severity score (ISS) was level 9 without any associated injuries. After 10 months, the patient evolved with varus consolidation, chronic osteomyelitis and migration of the proximal interlocking screws in different directions with the caudal screw migrating laterally (characteristic of the Z-effect). Because of the protruding inferior screw, it was necessary to remove the implant and to treat the chronic osteomyelitis. Radiographic images are presented in Figure 1.

### Case 2

**Figure 2:**
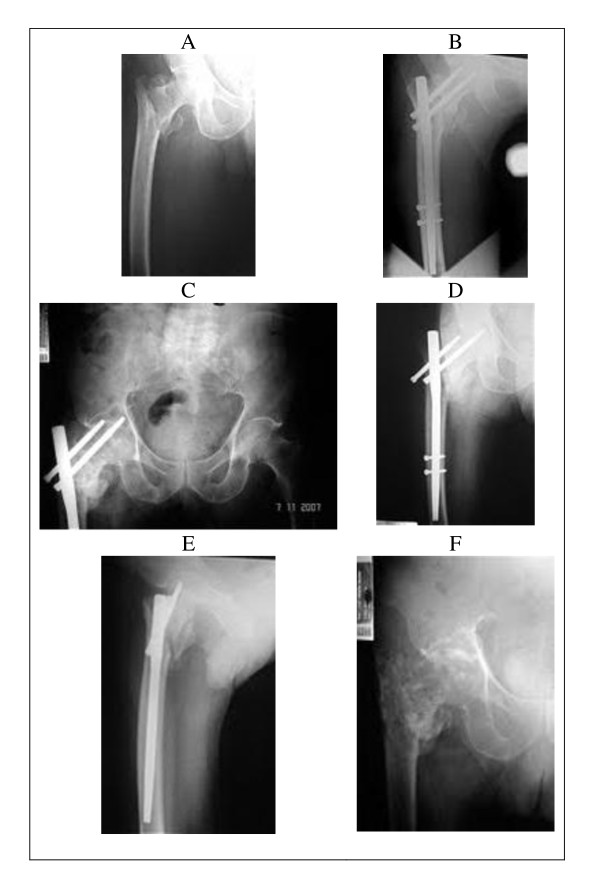
**Case 2 - Radiographic images showing the intertrochanteric fracture that was fixed with a proximal femoral nail** - (A) preoperative and (B) postoperative; (C and D) the lateral migration of the superior screw and perforation of the femoral head by the inferior screw (reverse Z-effect); (E) the incorrect positioning of the interlocking screws in the lateral incidence, together with poor bone material, may have been responsible for the reverse Z-effect; (F) the removal of the material of osteosynthesis is shown together with signs of chondrolysis of the hip.

A 68-year old retired male (a diabetic and well-controlled hypertensive patient) with a history of falling, presented intertrochanteric fracture of the right femur (AO/OTA 31-A2.2). The patient was submitted to osteosynthesis with PFN (Hexagon^®^) 36 hours after the trauma and evolved with migration of the proximal interlocking screws in opposite directions. The superior screw dislocated laterally, and varus collapse led to perforation of the femoral head by the inferior screw (reverse Z-effect). Since consolidation of the fracture had occurred, the implant had to be removed three years after osteosynthesis. The patient mobilized in the confines of his domicile with the aid of a walking stick. Radiographic images are presented in Figure 2.

### Case 3

**Figure 3:**
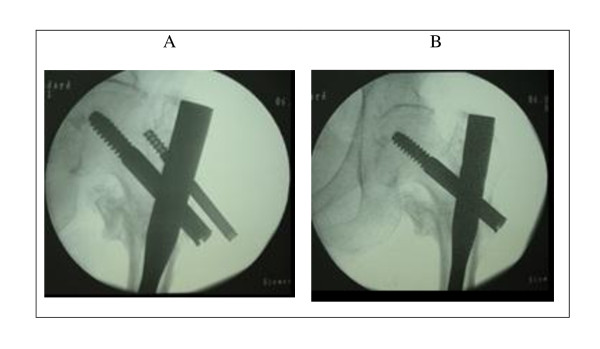
**Case 3 - Radiographies of the left hip joint showing the correct consolidation of the left intertrochanteric femoral fracture (A), together with the lateral migration of the superior screw while the sliding screw remained in the original position**. **The removal of the superior screw is shown in (B).**

An 80-year old housewife was injured in a fall resulting in lateral trauma of the left hip that was diagnosed as an intertrochanteric fracture of the left femur (AO/OTA 31-A2.1). The patient had a history of ischemic cardiomyopathy and had undergone angioplasty and vascular stenting, and was receiving a daily dose of Clopidogrel^® ^(75 mg). The patient was submitted to osteosynthesis with PFN (Synthes^®^, Rio Claro, SP, Brazil) one day after the trauma and evolved with fracture consolidation in a favorable position. After surgery, the patient returned to normal functional activities but the superior screw migrated laterally after 6 months. Although the inferior screw remained in the normal position the cranial screw migrated, probably owing to poor bone quality. The superior screw was removed after consolidation of the fracture. Since the sliding screw was well positioned with regard to the tip apex distance, and the fracture was adequately reduced, no failure in osteosynthesis occurred. Radiographic images are presented in Figure 3.

## Discussion

The treatment of proximal femoral fractures remains a challenge for trauma surgeons. The high rate of complications (> 30%), together with the large variety of techniques currently available, demonstrate that this problem has yet to be resolved satisfactorily [1].

Femoral proximal fractures are treated surgically with the dual objectives of avoiding clinical problems resulting from bed confinement and of providing the patient with a quality of life similar to that enjoyed pre-injury.

Surgical stabilization of the fracture is of crucial importance and can be achieved through the application of either intra- or extramedullary implants. There are, however, conflicting opinions regarding the type of implant that is most appropriate. For unstable fractures, intramedullary implants generally present biomechanical advantages over their extramedullary counterparts [2,3], and numerous studies have demonstrated satisfactory results following the use of such implants in the treatment of FPF. In contrast, some researchers have reported the occurrence of serious complications after using such procedures, including migration of the proximal screws and perforation of the femoral head, varus collapse of the fracture, cut-out, and fracture at or below the level of the terminus of the femoral nail [2-4].

The use of intramedullary implants comprising a single proximal interlocking screw results in significant rates of rotational instability and varus collapse of the fracture [5-7]. Additionally, complications involving fractures at or below the level of the terminus of the short femoral nail have been reported [8]. With the aim of increasing the stability of the fracture fixation, an anti-rotational screw has been introduced into the system. However, further complications such as the migration of screws in opposite directions (Z-effect and reverse Z-effect) and consequent varus collapse of the fracture have been described.

The Z-effect involves the lateral migration of the inferior screw, varus collapse of the fracture and perforation of the femoral head by the superior screw. The reverse Z- effect involves the lateral migration of the superior screw accompanied by the medial migration of the inferior screw. The first account of the Z-effect has been attributed to Werner-Tutshcku et al. [9], who reported a series of 70 cases of fractures treated using PFN. These authors also advised that fixation of the fracture at a cervico-diaphyseal angle of <125° is a predisposing factor for the Z-effect and reverse Z-effect, as well as for cut-out of the femoral head by the screw.

Although the literature describes the Z-effect and reverse Z-effect as the migration of proximal interlocking screws in opposite directions, in practice, sometimes only one screw actually migrates and the fracture undergoes an accommodation process that may lead to the perforation of the femoral head by the screw that remains in the normal position. Although the cause of this complication has been explained by varus collapse of the fracture and the lack of medial cortical support, its precise etiology requires further clarification [10].

Strauss et al. [10] have reproduced the migration of the cephalic screws from the intramedullary nail in the laboratory with the aid of a polyurethane model. These authors observed that when the compressive forces on the femoral head were greater than those on the femoral neck, the inferior screw migrated laterally. However, no reverse Z-effect was observed when the strengths of the respective forces were transposed. The finite element-based analysis used by these authors indicated that the differences in bone density at the locations where the two screws were fixed represented an important factor in understanding the independent performances of the screws. When the femoral neck presented a lower bone density than the femoral head, which is typical of unstable fractures, there was a tendency for the inferior screw to migrate. Strauss et al. [10] also suggested that the use of femoral nails comprising two interlocking head screws should be avoided in cases of fractures with intense comminution and loss of medial support.

In an attempt to solve this problem, implants have been devised that include specific safety features aimed at avoiding screw migration [11]. Additionally, novel devices incorporating helical blades that are introduced under impaction towards the femoral head, have been designed with the aim of increasing rotational stability, preserving the bone material of the femoral head and preventing varus collapse. Although such implants offer greater biomechanical stability in comparison with conventional PFN [11], they are not free of complications. Thus, Brunner et al. [1] have described three cases of perforation of the femoral head by helical blade devices in patients showing good fracture reduction and satisfactory positioning of the implant. These authors advise that in cases of severe osteoporosis, positioning of the blade at 5 mm or less below the joint should be avoided in order to prevent perforation of the femoral head.

In this paper, we report the unfavorable evolution of three patients undergoing osteosynthesis of proximal femoral fractures with intramedullary nails comprising two interlocking head screws. The retrospective analysis of these patients leads us to believe that the problems resulted from a series of factors including loss of medial support, varus collapse, inadequate entry point of the nail, and poor bone quality, or a combination of all such factors. Elucidation of the reasons for the occurrence of the reverse Z-effect observed in some types of fractures is still pending and requires further investigation.

## Conclusion

Although intramedullary fixation is an established method for the treatment of FPFs, it may evolve with complications including the migration of the interlocking head screws. Such an unfavorable evolution can be minimized by careful selection of the correct entry point of the nail and reduction of the fracture in order to avoid fixation in varus. For fracture patterns in patients presenting severe osteoporosis or medial comminution, the use of helical blades is recommended.

## Abbreviations

AIS: abbreviated injury scale; AO/OTA: Arbeitsgemeinschaft für Osteosynthesefragen/Orthopaedic Trauma Association; FPF: femoral pertrochanteric fractures; ISS: injury severity score; PFN: proximal femoral nail

## Ethics and consent

The Ethical Committees of the hospitals involved approved the study. Written informed consent to publish the case reports and the accompanying images was obtained from all of the patients concerned. Copies of the written consents are available for review by the Editor-in-Chief of this journal.

## Competing interests

The authors declare that they have no competing interests.

## Authors' contributions

RESP and DB were responsible for developing the idea of this study. RESP, EOSJ, LENS, VG, DB and FBR contributed to the study design. RESP, EOSJ and DB were involved in reviewing records and in data acquisition. RESP, EOSJ, VG and DB performed the literature review and drafting of the manuscript. All authors were involved in reviewing and editing the manuscript and all have approved the final manuscript.

## Authors' information

RESP obtained his Masters Degree at the Medical School of Universidade Federal de São Paulo. He is Assistant Professor in the Locomotor System Department of Universidade Federal de Minas Gerais.

EOSJ is a Medical Resident of the Orthopedic and Trauma Service of Hospital Felício Rocho.

LENS is Fellow Physician in the Hip Surgery Group of Hospital Felício Rocho.

VG obtained his Masters Degree at the Medical School of Universidade Federal do Rio de Janeiro and is Fellow Physician in the Trauma Group of Hospital Municipal Miguel Couto.

DB obtained his Masters Degree at the Medical School of Universidade Federal de São Paulo and is now a Fellow Physician of the Orthopedic Trauma Group at this Institute.

FBR obtained the Title of 'Livre Docente' and is a Professor at the Orthopedic and Trauma Department of Universidade Federal de São Paulo.
